# Management of a complex cholecystocutaneous fistula with laparoscopic retrieval of gallstones embedded in the abdominal wall

**DOI:** 10.1093/jscr/rjad619

**Published:** 2023-11-15

**Authors:** Jada Saunders, Panagiotis Kapsampelis, Tarak Chouari, Lawrence Nip, Ioannis N Gerogiannis

**Affiliations:** Department of General and Emergency Surgery, Kingston Hospital NHS Foundation Trust, Kingston Upon Thames, Greater London, KT2 7QB, United Kingdom; Department of General and Emergency Surgery, Kingston Hospital NHS Foundation Trust, Kingston Upon Thames, Greater London, KT2 7QB, United Kingdom; Department of General and Emergency Surgery, Kingston Hospital NHS Foundation Trust, Kingston Upon Thames, Greater London, KT2 7QB, United Kingdom; Department of General and Emergency Surgery, Kingston Hospital NHS Foundation Trust, Kingston Upon Thames, Greater London, KT2 7QB, United Kingdom; Department of General and Emergency Surgery, Kingston Hospital NHS Foundation Trust, Kingston Upon Thames, Greater London, KT2 7QB, United Kingdom

**Keywords:** cholecystocutaneous fistula, abdominal wall tract, cholecystitis, laparoscopic cholecystectomy, gallstones, Guillain–Barré

## Abstract

Cholecystocutaneous fistula is a rare surgical entity caused by an abnormal connection between the gallbladder epithelium and the skin. These complex cases have historically required an open surgical approach and are difficult to manage. We present a rare case of a 65-year-old male patient, with chronic lithiasic cholecystitis and cholecystocutaneous fistula. The patient underwent a laparoscopic subtotal cholecystectomy, dissection of the fistula tract, and removal of the impacted stones from the abdominal wall. With appropriate expertize, a completely laparoscopic approach is acceptable and the technical challenges can be predicted and overcome through careful pre-operative planning.

## Introduction

Cholecystocutaneous fistula (CCF) is an abnormal connection between the gallbladder epithelium and skin, first documented in the late 17th century [1]. The condition is rare, with <25 cases reported in the last 50 years [2–4]. Its incidence has significantly decreased owing to improved diagnosis and management of calculous cholecystitis, and nowadays cases represent the sequelae of neglected gallstone disease [5]. Other aetiologies include post-cholecystostomy complications [6], trauma, and gallbladder malignancy [7]. Open cholecystectomy with fistula tract excision is the most common surgical approach described [8]. The literature on laparoscopic cholecystectomy for this is limited and, there have been no reports in the literature of laparoscopic retrieval of gallstones impacted in the abdominal wall. We present a case of laparoscopic management of a complex CCF and the technical challenges associated with it.

## Case report

**Figure 1:**
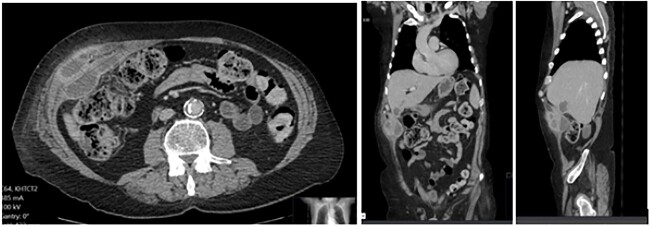
CT abdomen with portal venous contrast showing gallbladder collection extending to lateral abdominal wall.

**Figure 2:**
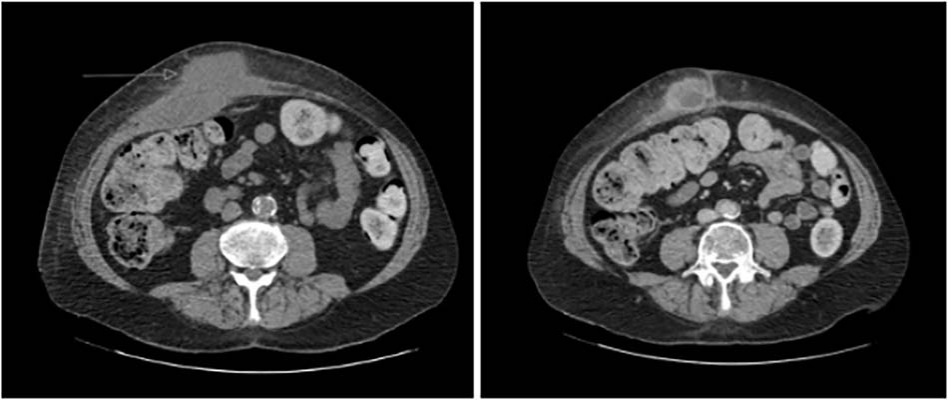
CT abdomen with portal venous contrast in axial plane showing extension of collection towards the paraumbilical region.

A 65-year-old gentleman with a body mass index of 29.6 and medical history of Guillain–Barré syndrome, type 2 diabetes mellitus, hypertension, and chronic pain was referred to our outpatient clinic with intermittent upper abdominal pain. His surgical history included an open appendicectomy. He was wheelchair-bound outside his home and received weekly assistance from a carer. On examination he was anicteric. His abdomen was soft, but a mildly tender mass was palpable in the right upper quadrant. An initial computed tomography (CT) scan showed thickening of the gallbladder and a collection extending from the fundus and infiltrating the anterior abdominal wall (Fig. 1). Further scans showed extension of the collection towards the midline (Fig. 2). He subsequently presented to the emergency department with purulent discharge from an external orifice adjacent to the umbilicus. An ultrasound-guided 6Fr pigtail drain was then placed into the known abdominal wall collection.

**Figure 3:**
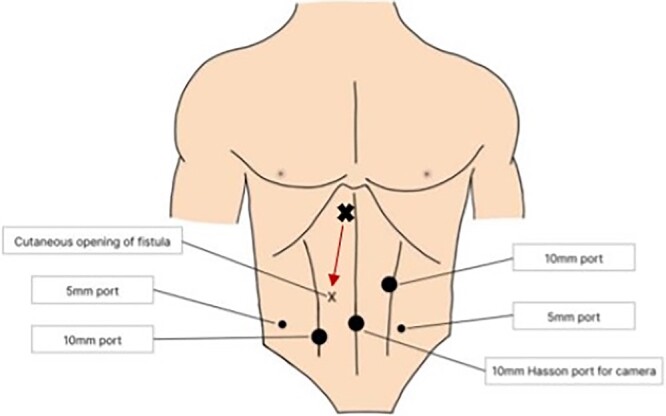
Location of laparoscopic ports to allow optimal dissection of gallbladder and fistula tract. The arrow shows the course of the fistula tract which originates from the gallbladder (**X**) just to the right of the falciform ligament and extends towards the cutaneous opening (x).

**Figure 4:**
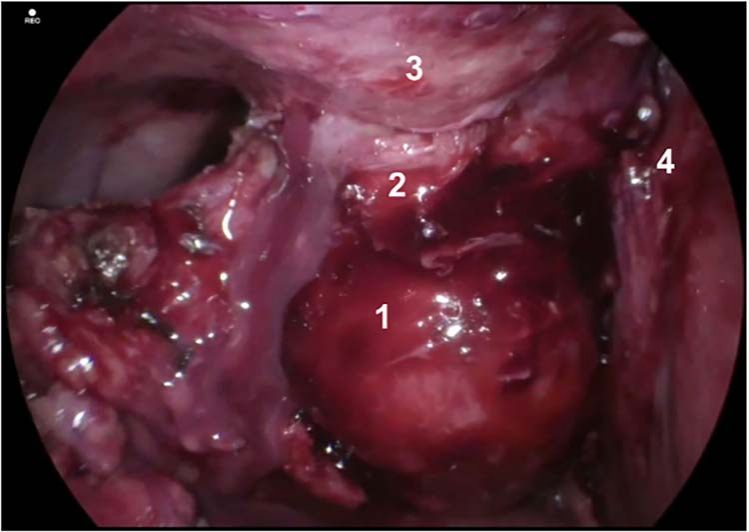
Gallbladder and fistula communicating with abdominal wall. Structures depicted: 1. Gallbladder, 2. Fistula, 3. Abdominal Wall, 4. Liver.

**Figure 5:**
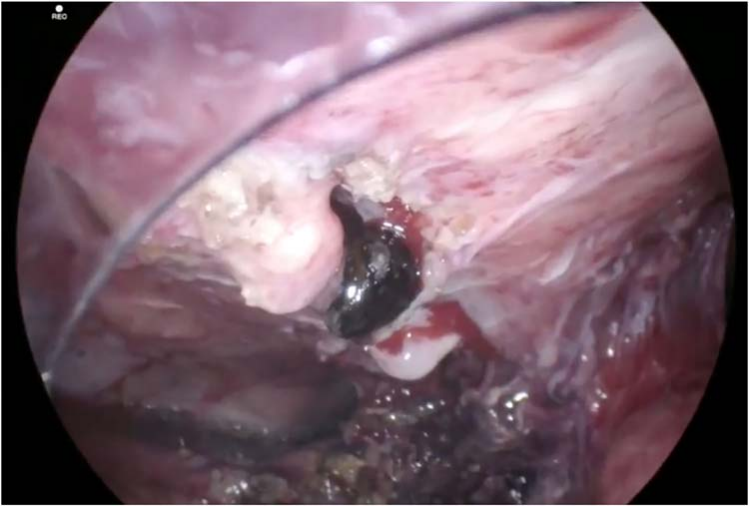
Internal opening of fistula tract after dissection of the gallbladder fundus away from the anterior abdominal wall. A gallstone is shown lodged in the fistula tract with further gallstones embedded more superficially.

Post-drain removal, the patient was booked for elective laparoscopic cholecystectomy. An infraumbilical port was inserted to achieve pneumoperitoneum, and further ports were inserted based on ergonomics (Fig. 3). Initial adhesiolysis and dissection of the gallbladder from the greater omentum and anterior abdominal wall proved challenging. After freeing the fundus, the gallbladder was found to be full of pigmented stones, many of which had migrated into the fistula tract (Fig. 4). The fistula tract was opened from the intra-abdominal aspect and all gallstones were laparoscopically retrieved, with some stones found to have migrated as far as the umbilical port in the midline (Fig. 5). A subtotal cholecystectomy was performed, leaving a small part of the Hartmann’s pouch in situ as it was unsafe to dissect lower. The pouch was closed with absorbable sutures. Drains were placed in the subhepatic space and pelvis.

The patient was transferred to the high dependency unit postoperatively. The subhepatic drain was monitored and noted to have a small amount of bilious output corresponding to a low volume bile leak from the gallbladder stump. This persisted; therefore, an endoscopic retrograde cholangiopancreatography with sphincterotomy and common bile duct stent placement was performed on post-operative Day 10. The patient was discharged on post-operative Day 14 with the subhepatic drain in situ and returned to the clinic two weeks later for its removal. Four years after the operation, the patient remains asymptomatic and the CCF is completely healed.

## Discussion

Due to its rarity, there are no international guidelines for the management of CCF. However, it is standard practice to treat with antibiotics and drain any associated abdominal wall abscess [8]. A systematic review of case reports found that the most common definitive treatment is open cholecystectomy and excision of the fistula tract [8]. There are a handful of cases reporting laparoscopic management of a CCF, but any stones impacted in the abdominal wall are removed with open surgery [6, 9–13]. This case represents the first description in the literature of the laparoscopic retrieval of gallstones which have migrated through a CCF tract and embedded in the abdominal wall.

Entering the abdomen necessitated pre-operative planning to locate a safe entry point devoid of visceral adhesions or fistulous tracts while maintaining ergonomic conditions. Our ports were inserted based on these principles. Port placement is important and should be placed at a sufficient distance from the gallbladder and fistula tract to allow adequate triangulation (Fig. 3). A bimanual technique with counter-pressure on the external opening was utilized to milk stones towards the internal opening. We acknowledge that limitations of this approach may exist with complex fistula tracts whereby stones may not be easily manipulated through the tract.

Due to its adherence to the anterior abdominal wall, conventional traction of the gallbladder is a significant technical challenge. In our case, the gallbladder was separated from the anterior abdominal wall early and a fundus-first approach was performed. Otherwise, it may be difficult to achieve the critical view of safety because of the inability to retract an immobile gallbladder when attempting to expose the hepatocystic triangle. A fifth port was placed in the left iliac fossa to further assist with retraction. During dissection across the CCF, dilation of the internal opening should be considered. Furthermore, the external opening of the tract may also need to be dilated, but this was not necessary in our case due to adequate drainage through the internal opening.

This case presented additional difficulties due to the presence of Guillain–Barré syndrome. Our patient required avoidance of non-depolarizing neuromuscular blockers and close control of his ventilatory capacity. Such patients should not be fully extubated until the respiratory and bulbar muscle reflexes have returned to normal, which may take some time [14]. Autonomic dysfunction can also cause wide swings in cardiovascular variables. It is therefore important to communicate clearly with the anesthetist regarding patient positioning or excessive blood loss, as these patients may overreact to changes in position and hypovolaemia.

In conclusion, CCF is a rare clinical entity and we have reported the first successful laparoscopic retrieval of gallstones migrating across the fistula tract. Our case demonstrates that laparoscopic cholecystectomy remains a safe option and that the technical challenges associated with it can be overcome with careful preoperative planning.

## Data Availability

For confidentiality reasons, we are unable to provide access to patient data. All other information should be requested from the corresponding author.
